# Development of 3D Slurry Printing Technology with Submersion-Light Apparatus in Dental Application

**DOI:** 10.3390/ma14247873

**Published:** 2021-12-19

**Authors:** Cho-Pei Jiang, M. Fahrur Rozy Hentihu, Yung-Chang Cheng, Tzu-Yi Lei, Richard Lin, Zhangwei Chen

**Affiliations:** 1 Additive Manufacturing Center for Mass Customized Production, National Taipei University of Technology, Taipei 106, Taiwan; fahrur@ntut.edu.tw (M.F.R.H.); kasu23@gmail.com (T.-Y.L.); 2 Department of Mechanical Engineering, National Taipei University of Technology, Taipei 106, Taiwan; 3 Graduate Institute of Manufacturing Technology, National Taipei University of Technology, Taipei 106, Taiwan; 4 Department of Mechatronics Engineering, National Kaohsiung University of Science and Technology, Kaohsiung 824, Taiwan; yccheng@nkust.edu.tw; 5 Department of Mechanical Engineering, The University of Auckland, Auckland 1142, New Zealand; rj.lin@auckland.ac.nz; 6 Additive Manufacturing Institute, Shenzhen University, Shenzhen 518060, China

**Keywords:** 3D slurry printing, submersion-light apparatus, zirconia, flexural strength, sintering, dental bridge, marginal-gap

## Abstract

This study proposes an innovative three-dimensional printing technology with submersion-light apparatus. A zirconia powder with an average particle size of 0.5 µm is mixed with 1,6-Hexanediol diacrylate (HDDA) and photo-initiator to form a slurry. The weight percentage of zirconia powder to HDDA is 70:30 wt.%. A light engine box is submerged in a slurry and emits a layered pattern to induce photopolymerization and transform a slurry into a printed green body. Green body sintering parameters for the first and second stages are 380 °C with a holding time of 1.5 h and 1550 °C with a holding time of 2 h. The sintered parts’ length, width, and height shrinkage ratios are 29.9%, 29.7%, and 30.6%. The ball milling decreases the powder particle size to 158 ± 16 nm and the mean grain size of the sintered part is 423 ± 25 nm. The sintered part has an average hardness of 1224 (HV), a density of 5.45 g/cm^3^, and a flexural strength of 641.04 MPa. A three-unit zirconia dental bridge also has been fabricated with a clinically acceptable marginal gap.

## 1. Introduction

Vat photopolymerization technology is classified according to the direction of the light projected onto the photocurable liquid resin: the top-down and bottom-up methods. The top-down method projects the layer pattern from the top to the surface of the liquid resin. Stereolithography (SL) is a top-down method of a three-dimensional additive manufacturing process that uses light to induce photopolymerization. It is used for three-dimensional (3D) printing of photocurable ceramic slurry. The photocurable ceramic slurry consists of ceramic powder and a photocurable resin. The content of the ceramic powder inside the ceramic slurry has a significant effect on the viscosity coefficient of the slurry. The higher the content of powder in the slurry, the greater its viscosity coefficient. Some 3D printing methods often use scrapers in the recoating layer process to eliminate surface tension in the recoated layer and agitate the slurry to inhibit the precipitation of powder particles [1]. An agitator is frequently added to a top-down type SL printer to ensure that the powder is suspended in the slurry.

The powder suspension capacity in slurry has a more significant effect on the success of a bottom-up 3D slurry printer. The shallow depth of the vat causes particles in the slurry to quickly sink to the bottom of the vat block light, which decreases the 3D printing success rate [2]. Therefore, increasing the powder suspension capacity for a slurry is a common field of inquiry in 3D ceramic printing [3]. A solvent-based slurry 3D printing system uses a solvent to adjust the viscosity coefficient of the slurry [4]. The most commonly used solvent is alcohol. The alcohol must evaporate from each new recoated layer during the 3D printing process before being exposed to light for photopolymerization. This method is only suitable for a top-down type slurry 3D printing system. Still, a significant disadvantage of this method is that the slurry cannot be reused, so there is increased material waste. Commonly used resin components in the ceramic slurry are 1,6-Hexanediol diacrylate (HDDA) and trimethylolpropane triacrylate (TMPTA) [5]. HDDA has a lower viscosity coefficient than TMPTA, so HDDA-based ceramic slurry is more suitable for a bottom-up photopolymerization slurry 3D printing than TMPTA-based ceramic slurry because it has a lower viscosity coefficient for the same powder content. However, the full content of 0.2 µm yttria-stabilized zirconia (YSZ) ceramic powder in the slurry is 40 vol.%, so the shrinkage ratio in the thickness direction is 28.6%, and there is non-linear shrinkage in the X-Y direction [6]. Increasing the content of ceramic powder in a slurry decreases shrinkage and increases the accuracy and the mechanical strength of a sintered part.

Sintering is a method of increasing the density of a printed ceramic object. Still, improper sintering causes cracks [7], shape distortion [8], and delamination [9], which produce undesired and unpredictable mechanical properties. The greater the content of ceramic powder in the printed ceramic object, the smaller the shrinkage rate for the sintered part. Theoretically, the lower the density of the powder particles, the greater the powder’s content in the slurry and the smaller the shrinkage rate. Therefore, the density of a ceramic powder is one of the influencing factors for shrinkage rate.

Studies of 3D ceramic slurry printing mainly optimize the ratio of powder content to photocurable resin to achieve the highest powder content in ceramic slurry and ensure the highest 3D printing success rate. The ceramic powders that are used for 3D slurry printing include alumina (Al_2_O_3_) [10,11], with a density of 3.95 g/cm^3^, and zirconia (ZrO_2_) [12,13], with a density of 5.68 g/cm^3^. 3D zirconia slurry printing can be used to fabricate bone grafts [14,15] and permanent dental restoration devices [16,17]. Studies show that the maximum content of zirconia powder in the slurry is 60 wt.%, giving a maximum shrinkage rate of 35.25% after sintering at 1500 °C with a holding time of 1 h [18]. One study shows that a 4 mol.% yttria partially stabilizes zirconia (4Y-PSZ) slurry with a powder content of 50 vol% to give a shrinkage rate of 18.8% after sintering at 1500 °C with a holding time of 2 h [19]. Current studies of 3D ZrO_2_ slurry printing maximize the content of zirconia powder in the slurry, control the shrinkage rate, and increase the 3D printing success rate. Most 3D ZrO_2_ slurry printing studies fabricate a single crown because the volume is small, and it is easier to control shrinkage than for a three-unit dental bridge [20,21]. This study fabricates a three-unit dental bridge model with a clinically acceptable marginal gap as a benchmark to demonstrate the feasibility of the proposed process for a dental application.

This study proposes an innovative submersion-light 3D slurry printing process that increases the suspension capacity for powder in a ceramic slurry. A double-sided constraint method for the cured layer combines the advantages of the top-down type and the bottom-up type photopolymerization methods. The ceramic material is zirconia powder. A zirconia slurry is produced with a maximum powder content that allows 3D printing. Two-stage sintering is used, and the sintering parameters are determined according to thermogravimetric analysis results (TGA). The sintering shrinkage ratio for the printed parts is determined. A three-unit dental bridge is fabricated as a benchmark to assess its suitability for clinical use.

## 2. Materials and Methods

### 2.1. Submersion-Light Three-Dimensional Slurry Printing Method

**Figure 1:**
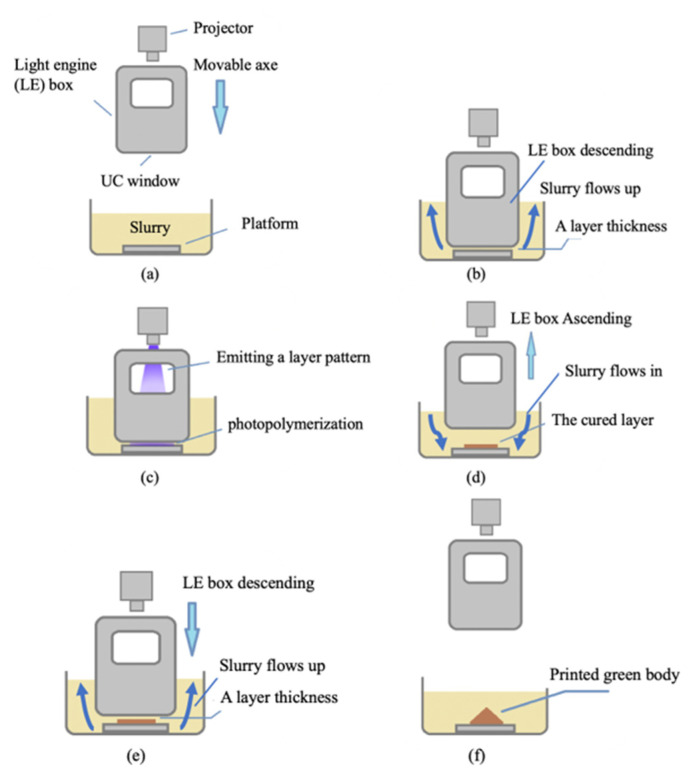
(**a**–**f**) The principle of the submersion-light 3D slurry printing method.

Most of the vat photopolymerization in the market uses a steady light source and moving build platform. A printed product that sticks on a moving build platform might split out from the build platform and cause a failed printing. This happens especially in the bottom-up method. Separation force/peeling force between printing product and vat surface is a common reason for the failure [22]. To lower the possibility of this failed printing, a novel submersion-light printing method is proposed. This method reverses the bottom-up approach. The build platform that usually moves up and down layer by layer becomes steady; on the other hand, the light source that is typically steady becomes moving up and down, layer by layer. Not just moving, the light source is submerged into the vat. Figure 1 shows the printing method for the proposed process of 3D printing with submersion-light apparatus. It uses a movable projector with an upper constraint (UC) window as a light engine (LE) box, as shown in Figure 1a. The UC window consists of highly transparent glass and a soft interface for departing. The platform is mounted on the bottom of the slurry vat. Figure 1b shows that the slurry flows when the LE box submerses itself in the slurry, and the UC window maintains the layer thickness distance between the platform. The LE box emits the layer pattern transmitted by light with a wavelength of 405 nm onto the slurry between the UC window and the platform to induce photopolymerization, as shown in Figure 1c. The cured layer bonds with the platform and adheres to it. At the same time, the LE box ascends because the soft interface on the UC window allows an elastic deformation to depart, and the gravity of the cured layer causes separation, as shown in Figure 1d. As the LE box ascends, the slurry flows, so the ceramic powder is suspended, and the slurry does not separate or form sediment during the printing process. Figure 1e shows the LE box submerged in the slurry. The slurry flows upward, and a layer thickness distance is maintained for the next layer of printing. The green body is produced by repeating (e), (c), and (d) until all layers are printed, as shown in Figure 1f.

### 2.2. Mechanical Design of the Proposed Printer

**Figure 2:**
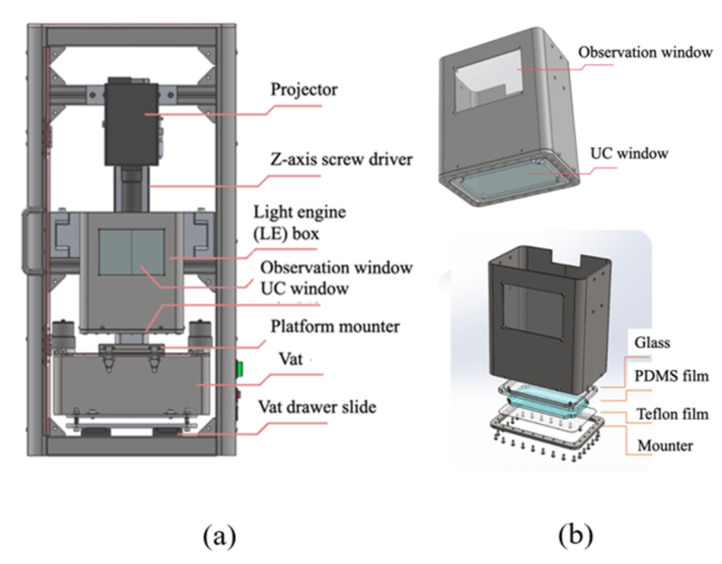
The mechanical design of the proposed printer (**a**) and the disassembled components of the LE box (**b**).

A printer must feature good expandability, a small size, ease of installation for the slurry vat, and observe the layer curing status. Figure 2a shows the proposed design. A lead screw with a resolution of 2 µm is the *Z*-axis movement that moves the LE box steadily and vertically. The projector, which is also called a light engine, is placed on the *Z*-axis lead screw. The light engine uses a light-emitting diode with a wavelength of 405 nm and a power intensity of 4.25 watt/cm^2^. The working distance of the lens is 184 mm because the effective exposure area is 131 mm × 80 mm for a resolution of 100 µm. The LE box is mounted on the *Z*-axis lead screw and beneath the projector to maintain a distance equal to the projector’s focal length. The slurry vat is placed at the vat drawer slide. Figure 2b shows the LE box with observation and UC windows. The status of the curing layer is observed through the observation window. The UC window is constructed of glass, a PDMS film, a Teflon film, and a mounter. Both films allow soft elastic deformation, which creates a tiny void resulting in the departing of the cured layer, so the cured part bonds with the platform. The use of PDMS film has been proven to reduce the pull-up force of the platform after the curing process [23].

### 2.3. Material Preparation

**Figure 3:**
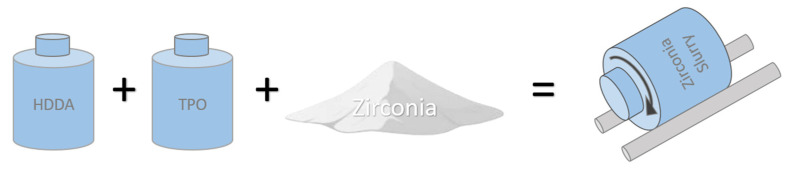
Preparation of making zirconia slurry.

The light-curable zirconia slurry consists of zirconia powder, resin, and photo-initiator. Zirconia powder is preferable to other ceramic powder such as alumina because of the high flexural strength and better fracture resistance [24] and especially the aesthetic factor of zirconia color that is more similar to natural teeth [25]. The photo-initiators used in this study are TPO (Doublecure TPO, Double Bond Chemical Ind., Co., LTD, Taipei, Taiwan). The absorbance wavelengths are 350~410 nm, which corresponds to the emission wavelength of the light engine. HDDA is used as resin because it has a lower viscosity than TMPTA. The powder is 3 mol.% yttria-stabilized zirconium-dioxide (3YSZ-7, Zipro Co., Taipei, Taiwan), with an average particle size of 0.5 µm. The proportion of zirconia powder in the slurry was increased during the test because it directly affects the viscosity of the slurry. Slurry preparation is shown in Figure 3. The content ratio of zirconia (ZrO_2_) powder to resin for this study is 50:50 wt.%, 60:40 wt.%, and 70:30 wt.%. The proportion of photo-initiator is fixed at one wt.% of the resin amount. The slurry was prepared by placing ZrO_2_ powder, resin, photo-initiator, and zirconia balls in specific proportions in a light-shielding bottle, and a rolling ball machine was used to achieve a slurry with a uniform mixture and to grind the powder granules to less than 200 nm.

### 2.4. Physical Property Measurement

#### 2.4.1. Viscosity

As the LE box ascends, a shear force is produced between the UC window and the cured layer, which can damage the cured layer. The faster ascension and the larger the viscosity coefficient, the greater the shear force generated. The viscosity coefficient was measured to optimize the ascension speed of the LE box. The viscosity coefficient of the slurry was measured using a viscosity tester (Brookfield DV3T) at room temperature. Two rotors are used. The viscosity of the slurry was unknown, so the CPA-40Z rotor was used for low viscosity, and the CPA-52Z rotor was used if the viscosity of the slurry exceeded the viscosity range that CPA-40Z can measure.

#### 2.4.2. Slurry Curing Test

**Figure 4:**
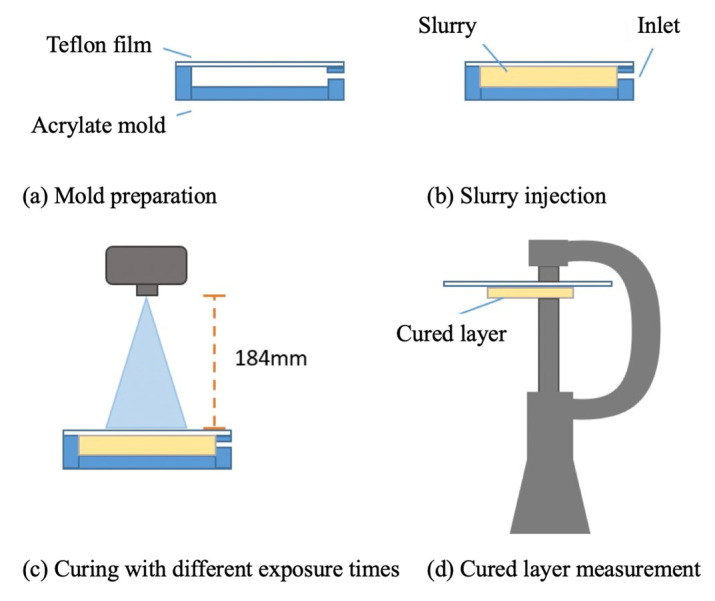
The measurement procedure for the slurry curing test. (**a**) Mold preparation; (**b**) Slurry injection; (**c**) Curing with different exposure times; (**d**) Cured layer measurement.

Incident light is scattered between the powders in the ceramic slurry, so the cured layer is wider and shallower than the pure resin. The greater the powder content, the more energy attenuated, so the slurry curing test measured the cured depth in terms of the exposure to light energy. Figure 4 shows the proposed procedure for the curing test in this study, which used an acrylate mold with a Teflon film, as shown in Figure 4a. The slurry was injected into the mold cavity and exposed to light exposure for various periods, as shown in Figure 4b and Figure 4c respectively. The cured layer was measured using a micrometer caliper with a resolution of 1 µm, as shown in Figure 4d.

#### 2.4.3. Microstructural Observations and Mechanical Properties

The microstructure of a sintered ZrO_2_ part was measured to ensure a stable phase structure and to calculate the grain size. Studies show that the grain size of ZrO_2_ increases as the fracture strength increases [26] and as the hardness decreases [27]. This study uses electric scanning microscopy (SEM, JSM-7610F, Tokyo, Japan) to measure the particle size of the as-received powder and the grain size of the sintered part. Hardness test is based on ASTM C1327-03 using Shimadzu HMV-G21S Vickers hardness test machine, and three-point bending test is based on ASTM C1161-13 using Universal Testing Machine (Chun Yen, Taiwan) CY-6102.

Archimedes’ principle was used to measure the density of the sintered part. The dimensions of the sintered part were 10 mm × 10 mm × 10 mm. The weight of the sintered part was measured in air ($W_{air1}$), and then it was soaked in water, the surface was wiped, and the weight of the sintered part was measured again in air ($W_{air2}$). The sintered part was then immersed in a tank of pure water, and the weight of the tank was measured ($W_{water}$). The density is calculated using Equation (1):

(1) $$density\left(D\right)=\frac{W_{air1}\times water\;density}{W_{air2}-W_{water}}$$

#### 2.4.4. Thermogravimetric Analysis and Two-Stage Sintering

The parameter settings for the two-stage sintering treatment must be optimized to ensure that the sintered part has no cracks and sufficient strength. The slurry uses HDDA as a resin, TPO as a photo-initiator, and zirconia powder. The printed green body is formed when the photocurable resin absorbs photons and polymerizes the resin and powder. The temperature for the first stage must be sufficient to vaporize the polymerized resin. The dwell time for the first stage must ensure that the resin is completely evaporated. The temperature was increased and held for the second stage at the temperature at which the zirconia powder fuses and crystallizes. The crystal growth achieved a sufficiently high-density zirconia strength.

A thermogravimetric test (TGA) was used to determine the temperature for the first stage of the sintering. Hitachi STA 7300 with air atmosphere is a device that is used for the TGA test. The sintering temperature of 1550 °C is for the second stage. The holding time for the second stage was 2 h to allow the growth of differently sized grains to determine the effect of grain size on hardness and flexural strength.

#### 2.4.5. Shrinkage Measurement

In the printed green body, the resin is vaporized during the first stage of sintering. The powder is fused to produce crystals, so the density at the second-stage sintering temperature increases. This process causes volume shrinkage. The difference between the volume of the printed part before and after sintering must be measured along each axis to increase the accuracy of the ceramic sintered part. An ideal printed green body must feature an approximately linear shrinkage rate in each direction to compensate for the magnification of the printed model. Therefore, this study prints six cubic specimens with dimensions of 10 mm × 10 mm × 10 mm and sinters them to determine the shrinkage ratio. A caliper is used to measure the length along each axis.

### 2.5. Benchmark Fabrication

**Figure 5:**
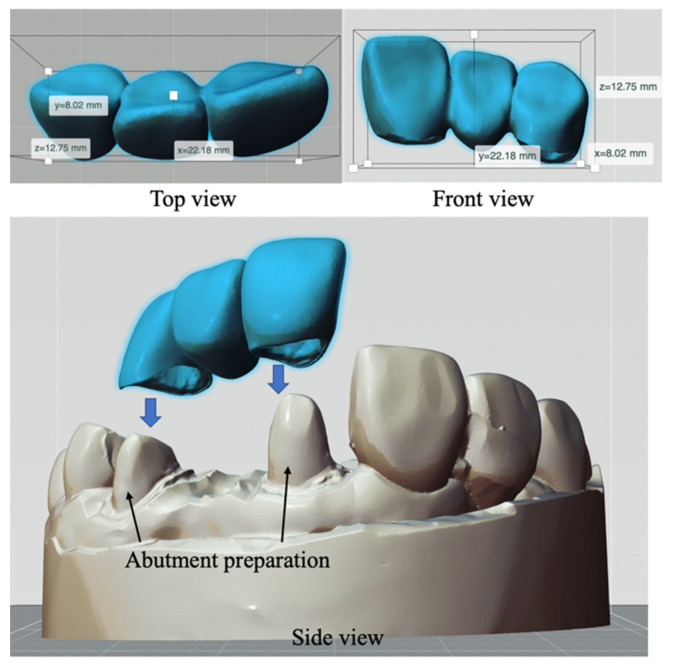
The proposed benchmark: top and front views (**top**) and assembled view for a three-unit dental crown and a dental stone model (**bottom**).

A benchmark three-unit dental bridge was printed using zirconia green bodies, as shown in Figure 5. The dimensions in the X, Y, and Z directions are 22.18 mm × 8.02 mm × 12.75 mm. The sintered parts must fit the marginal abutment line for dental stone models, so the sintered parts were mounted on the stone model and reverse scanned to determine the gap between the sintered part and the marginal line. Some researchers have also done a marginal gap measurement [28,29] which is the gap (distance) between the crown and the teeth gums, as the fit evaluation analysis.

## 3. Results and Discussion

### 3.1. Development of the Submersion-Light Three-Dimensional Slurry Printer

**Figure 6:**
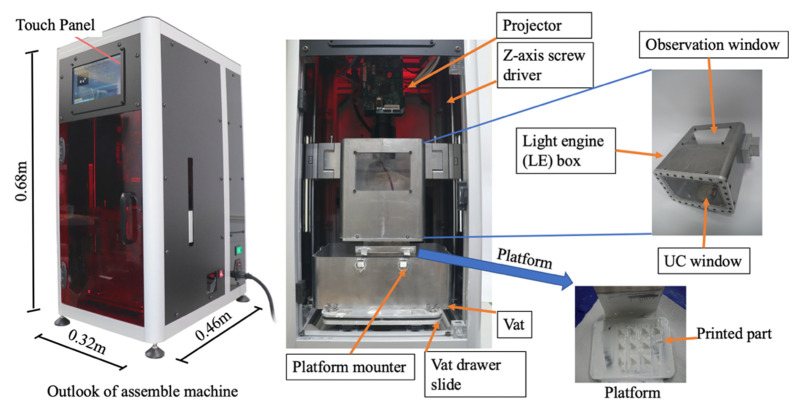
The assemble machine and all moduli.

Figure 6 shows the assembled machine. The projector is located above the LE box and synchronizes with the LE box at a fixed-focus distance. The vat was pulled out to locate the platform on the bottom of the vat. The slurry was poured into the vat, and the vat was returned to its original position. Figure 6 also shows that the LE box has an observation window and a UC window. When the sliced layer is imported into the control interface, the printer is ready for printing. During the printing process, the user observes the layer projection pattern through the UC window to determine the success of the cured layer. After printing, the platform was removed from the vat and cleaned to achieve the printed green bodies.

### 3.2. Viscosity Measurement

Three types of slurry were produced with ratios of ZrO_2_ powder to the resin of 50:50 wt.%, 60:40 wt.%, and 70:30 wt.%. The viscosity was then measured. The experimental results show that the 50 wt.% and 60 wt.% ZrO_2_ slurries have low viscosity, so the CPA-40Z rotor was used for measurement. The CPA-52Z was used to measure the 70 wt.% ZrO_2_ slurry because this exceeds the viscosity range for the CPA-40Z.

**Figure 7:**
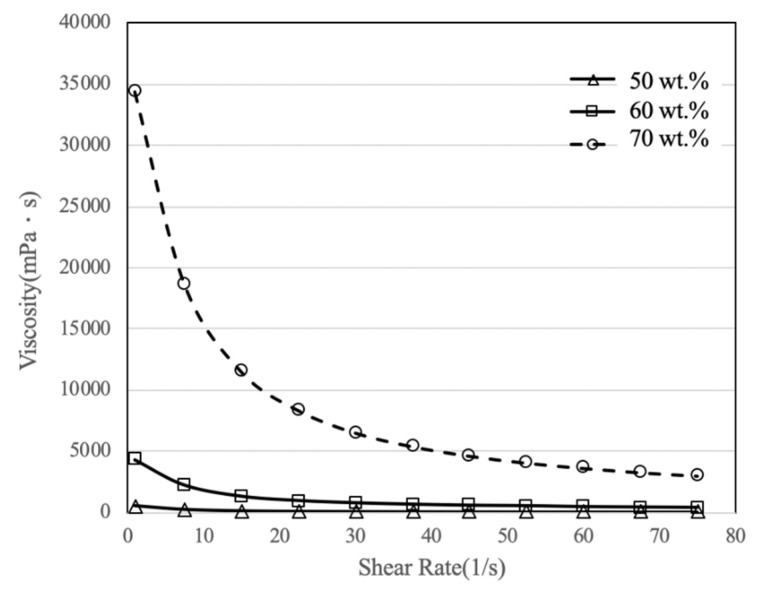
Viscosity curves for three types of zirconia slurry.

The relationship between the viscosity and the shear rate of zirconia slurry for these three ratios is shown in Figure 7. The shear rate increases as the viscosity coefficient of the slurry decreases. The 70 wt.% ZrO_2_ slurry has the highest viscosity. The relationship between the viscosity coefficient and the shear rate is known. Still, it does directly correspond to the ascension speed of the LE box, so the optimal lifting speed for the LE box must be determined experimentally.

### 3.3. Curing Results

**Figure 8:**
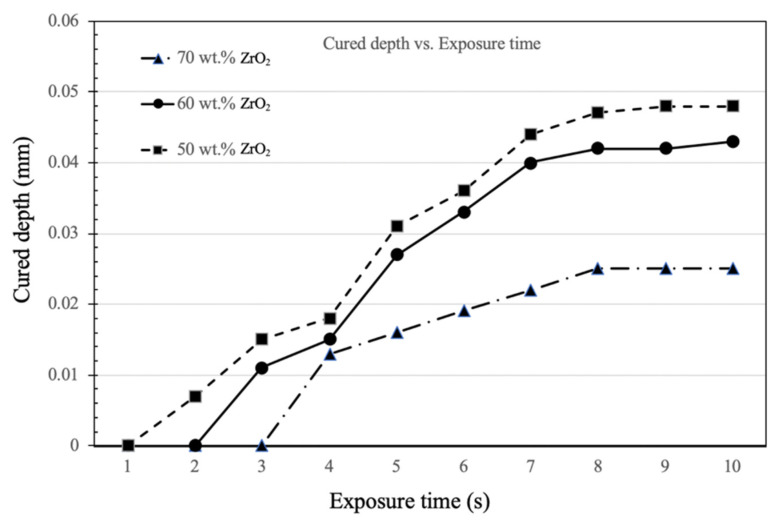
Curing depth curve for various exposure times (seconds).

The curing test used a power intensity of 4.25 watts/cm^2^. Figure 8 shows the relationship between exposure time and cured depth. For an exposure time of 7 s, the cured depth for 70 wt.% ZrO_2_ slurry is more than 0.02 mm, which is approximately half of the depth for the others. The cured depth for all slurries stops increasing when the exposure time exceeds 8 s, possibly because the cured layer reflects light, so it does not penetrate deeper and induce photopolymerization.

### 3.4. Optimizing the Ascension Speed of the LE Box

**Figure 9:**
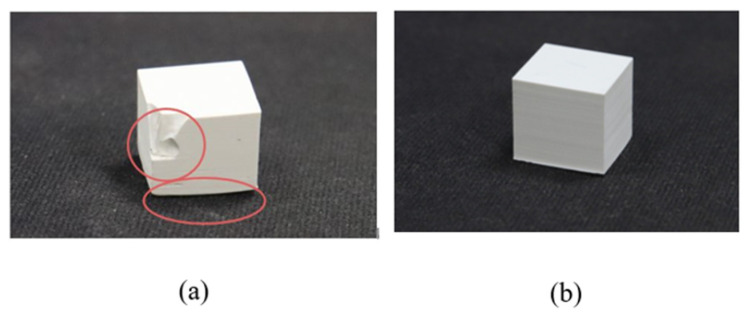
The printed cubic model using 70 wt.% ZrO_2_ slurry with the LE box ascending at 0.4 mm/s (**a**) with some cracks failure and 0.25 mm/s (**b**).

Several cubic specimens were printed to optimize the printing parameters. The layer thickness is 20 µm, and the exposure time is 8 s. The experimental results show that the cubic model is fabricated when the LE box ascends at 1 mm/s for 50 wt.% ZrO_2_ slurry and 0.8 mm/s for 60 wt.% ZrO_2_ slurry. However, the 70 wt.% ZrO_2_ slurry often features cracks at the cubic edge in the direction in which layers are constructed if the LE box ascends at 0.4 mm/s, as shown in Figure 9a, but a cubic model is printed successfully if the LE box ascends at 0.25 mm/s, as shown in Figure 9b. The optimal printing parameters for 70 wt.% ZrO_2_ slurry are an exposure time of 8 s, a sliced layer of 20 µm, and the LE box ascending at 0.25 mm/s.

### 3.5. Measurement of the Physical Properties

**Figure 10:**
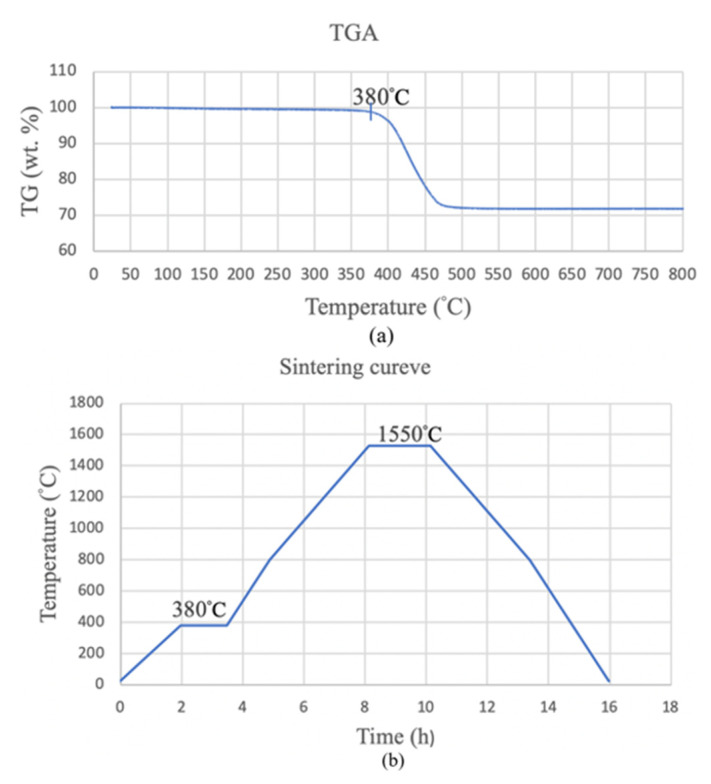
The results of the thermogravimetric analysis (**a**) and the two-stage sintering curve (**b**).

Figure 10a shows that the green body loses weight on the TGA curve at 380 °C. Maintaining this temperature for a period of time allows the resin to vaporize fully. Therefore, the temperature for the first stage is 380 °C, with a heating rate of 3 °C/min and a holding time of 90 min to allow all of the resin to evaporate. The temperature was increased to 1550 °C for the second stage, with a heating rate of 3.75 °C/min and a holding time of 2 h. The specimen was then cooled to room temperature in the furnace, as shown in Figure 10b.

### 3.6. Measurement of the Shrinkage Ratio

**Figure 11:**
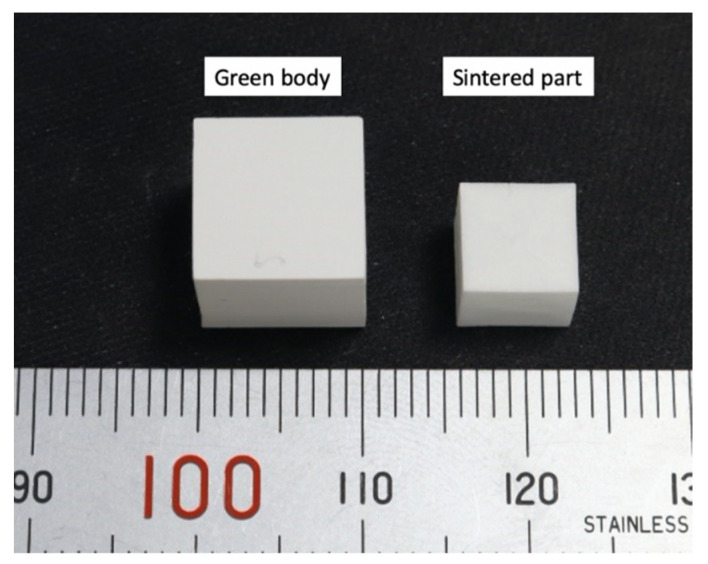
The difference in the dimensions of the printed green body (**left**) and the sintered part (**right**).

The cured resin vaporizes while the printed green body is sintered at the first stage, and the powder particles are fused, so the density is greater at the second stage of sintering. The sintered part is smaller than the green body, so the difference in size before and after sintering must be measured to control the size of the highly dense ceramic part. The enlarged magnification ratio for the printed green body is calculated using the shrinkage ratio in each direction to allow precise dimensional control of the highly dense ceramic part. Figure 11 shows the difference in the dimensions of the green body, which is 10.03 mm × 10.00 mm × 9.88 mm, and the sintered part, which is 7.02 mm × 7.04 mm × 6.87 mm. Six cubic green bodies were printed and sintered. The experimental results show that the average respective linear shrinkage rate is 29.9%, 29.7%, and 30.6% in the (X), width (Y), and height (Z) directions. This reveals that the shrinkage rate is approximately linear in each direction.

### 3.7. Microstructure and Mechanical Properties

The density after sintering depends on the sintering temperature. The theoretical density of ZrO_2_ is about 5.85~6.09 g/cm^3^, and the theoretical average density is 5.94 g/cm^3^. The sintered part has an average density of 5.45 g/cm^3^, 91.8% of the average theoretical density.

**Figure 12:**
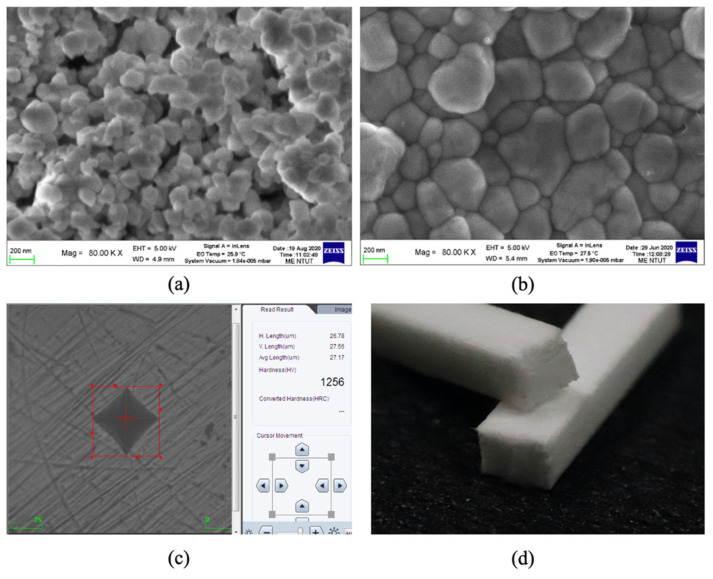
Microstructure of the printed green body (**a**), the sintered part (**b**), one of the indentation points for the hardness test (**c**), and fractured pieces for the three-point bending test (**d**).

Figure 12a and Figure 12b respectively show the SEM images of the as-printed green body and the sintered part. Both images show that the mean powder size in the printed green body after ball milling is 158 ± 16 nm and the mean grain size for the sintered part is 423 ± 25 nm. Figure 12b shows densely packed submicron-grade grains. Figure 12c shows the indentation point for the hardness measurement. According to ASTM C1327-15, a hardness test was conducted for three sintered parts using six indentation points on each piece. The average hardness is 1224 ± 26 HV (11.72 GPa). Figure 12d shows a photograph of a fractured sintered part after a three-point bending test. The mean flexural strength is 641.04 ± 82.12 MPa. The mean flexural strength was taken according to ASTM C1161-18 with 2 mm × 1.5 mm × 25 mm specimen dimension, and a total of 8 samples were tested to get the result.

### 3.8. Benchmark Fabrication

**Figure 13:**
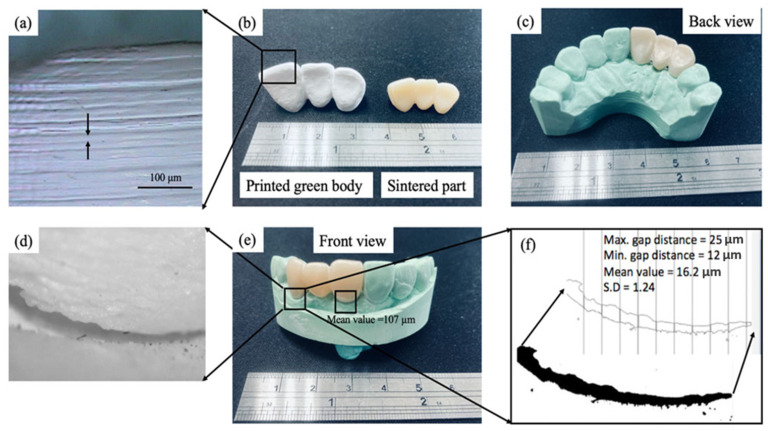
(**a**–**f**) The printed green body and the sintered part for the benchmark: micro observations and macro observations.

Figure 13b shows the printed green body and the sintered part for the benchmark. Figure 13a shows the microscope image. It shows that layer thickness is about 20 µm, and all layers bonded well without delamination. The sintered part was inserted into the dental stone, and the back view is shown in Figure 13c and the front view in Figure 13e. The marginal gap is the most indicator of accuracy for clinical use. The smaller gap is preferable for clinical use because of less gingival irritation [28]. A clinically acceptable marginal gap is less than 120 µm [30]. Using a milling process to produce a single crown gives a marginal gap of less than 80 µm [31]. One study compared the accuracy of a single crown produced using milling and 3D slurry printing and showed that both fabrication methods are equally accurate [11,32]. However, few studies fabricate a three-unit dental bridge using ZrO_2_ slurry printing and measure the marginal gap. Figure 13d shows the image of the marginal gap. The mean value for the marginal gap is 16.2 µm as shown in Figure 13e. However, the mean value for another side tooth is 107 µm. This indicates that sintering induces slight distortion because the middle tooth is solid, but both sides are hollow to allow insertion into an abutment. This specimen meets the marginal gap’s clinical requirement. Still, the flexural strength is less than the clinical requirement (800 MPa) for ISO 6872:2015 [33], so it opens more research opportunities to be used at dental restoration devices.

## 4. Conclusions

This study used a novel 3D slurry printing with submersion-light apparatus to fabricate the green body for a three-unit dental bridge. A slurry with 70 wt.% of zirconia powder and 30 wt.% of photocurable resin was synthesized. An LE box, consisting of a projector with a fixed-focus distance, was submerged into the liquid slurry, and the sliced layer pattern was emitted to induce photopolymerization. The optimal printing parameters were a layer thickness of 20 µm, an exposure time of 8 s, and the LE box ascending at 0.25 mm/s. The respective shrinkage rate for length, width, and height was 29.9%, 29.7%, and 30.6%. The sintered part had an average hardness of 11.72 GPa and a mean flexural strength of 641.04 ± 82.12 MPa. The marginal gap of the fabricated three-unit zirconia dental bridge was clinically acceptable.

For future works, some of the machine disadvantages, such as the heavier Z-Axis compared with the traditional system and the bigger UC window compared to a 3D platform in the conventional method, which creates more cleaning up work to be done after the UC window is submerged into the VAT, are some of the disadvantages that need to be considered as a challenge to improve the system.

## Data Availability

Not applicable.
